# Supplementary material for: Detection and Control of Dermatophytosis in Wild European Hedgehogs (Erinaceus europaeus) Admitted to a French Wildlife Rehabilitation Centre
Source: J Fungi (Basel). 2021 Jan 21;7(2):74. doi: 10.3390/jof7020074 (PMC7911743; doi:10.3390/jof7020074)
Supplement: Supplementary file 1 [file jof-07-00074-s001.pdf]

Supplementary Data.

Table S1: European hedgehogs (*Erinaceus europeaeus*) admitted at the rescue centre (ChuvFS Alfort) during 2016 and sampled for dermatophyte detection.

N°	ID	Date of arrival	Postal code	Outcome	Weight at arrival (g)	Sex	Skin lesions	1st sampling date	Sample ID	1st diagnosis	N° of samplings
1	**40**	17/01/2016	93160	R	550	female	no	17/01/2016	T256	negative	1
2	**64**	12/07/2016	77400	D	NA	NA	no	25/01/2016	T224	negative	1
3	**86**	13/07/2016	60940	R	154	female	no	01/02/2016	T254	negative	1
4	**122**	13/07/2016	60940	R	177	female	no	13/02/2016	T255	negative	1
5	**123**	13/07/2016	78340	D	585	female	no	13/02/2016	T258	positive	2
6	**186**	03/09/2016	NA	R	145	male	no	04/03/2016	T311	negative	2
7	**216**	03/09/2016	NA	R	315	male	no	11/03/2016	T412	positive	1
8	**281**	01/02/2016	94800	D	319	female	no	25/03/2016	T413	positive	3
9	**296**	01/05/2016	95260	R	1711	NA	yes	29/03/2016	T450	negative	2
10	**300**	01/07/2016	77185	D	540	female	no	30/03/2016	T449	negative	2
11	**319**	01/09/2016	94000	D	133	female	no	03/04/2016	T446	negative	1
12	**320**	01/12/2016	78640	E	291	female	no	03/04/2016	T447	negative	1
13	**322**	02/05/2016	95100	D	511	male	no	03/04/2016	T445	negative	1
14	**324**	02/06/2016	92700	D	402	male	no	04/04/2016	T444	negative	2
15	**364**	02/07/2016	78840	R	68	NA	no	08/04/2016	T493	negative	1
16	**388**	02/07/2016	78840	D	70	NA	no	12/04/2016	T494	negative	1
17	**390**	02/07/2016	95640	D	111	NA	no	12/04/2016	T508	negative	1
18	**393**	02/07/2016	95640	D	112	NA	no	13/04/2016	T507	negative	1
19	**403**	02/07/2016	95640	R	127	male	no	15/04/2016	T519	negative	2
20	**404**	02/07/2016	93160	D	156	NA	no	15/04/2016	T520	negative	2
21	**415**	02/07/2016	93360	R	629	female	no	17/04/2016	T521	negative	1
22	**418**	02/08/2016	75019	D	477	female	no	18/04/2016	T576	positive	1
23	**427**	02/09/2016	77163	D	193	male	no	19/04/2016	T572	negative	1
24	**441**	02/09/2016	77860	E	NA	NA	no	22/04/2016	T574	negative	2
25	**446**	02/10/2016	78500	NA	154	NA	no	23/04/2016	T573	negative	2
26	**454**	02/10/2016	94170	D	373	NA	no	25/04/2016	T575	positive	3
27	**472**	02/11/2016	94170	D	330	male	no	26/04/2016	T612	negative	1
28	**489**	02/12/2016	NA	D	300	female	no	28/04/2016	T613	positive	2
29	**501**	03/03/2016	91400	R	651	male	no	29/04/2016	T614	positive	1
30	**503**	03/04/2016	77680	D	260	NA	no	29/04/2016	T615	positive	1
31	**530**	03/04/2016	94190	R	472	NA	no	01/05/2016	T618	positive	2
32	**533**	03/04/2016	94800	D	758	female	no	02/05/2016	T620	negative	1
33	**555**	03/05/2016	94260	D	387	female	no	03/05/2016	T634	negative	1
34	**582**	03/06/2016	94410	E	NA	NA	no	06/05/2016	T635	negative	1
35	**606**	03/07/2016	95570	D	73	NA	no	08/05/2016	T657	negative	1
36	**616**	03/07/2016	NA	R	119	male	no	08/05/2016	T656	negative	1
37	**656**	03/07/2016	91300	D	202	NA	no	12/05/2016	T736	positive	2
38	**671**	03/08/2016	92170	R	203	female	no	13/05/2016	T733	negative	2
39	**675**	03/08/2016	91330	E	720	female	no	14/05/2016	T739	negative	1
40	**715**	03/08/2016	91330	E	NA	female	no	15/05/2016	T735	negative	1

N°	ID	Date of arrival	Postal code	Outcome	Weight at arrival (g)	Sex	Skin lesions	1st sampling date	Sample ID	1st diagnosis	N° of samplings
41	**763**	03/09/2016	94000	NA	116	female	no	17/05/2016	T742	negative	1
42	**766**	03/09/2016	77600	NA	143	NA	no	18/05/2016	T745	negative	1
43	**769**	03/09/2016	94500	D	190	female	no	18/05/2016	T741	negative	1
44	**818**	03/09/2016	92320	R	722	male	no	21/05/2016	T732	negative	2
45	**832**	03/10/2016	94130	NA	157	female	yes	22/05/2016	T747	negative	1
46	**873**	03/11/2016	NA	D	juvenile	male	no	24/05/2016	T804	positive	4
47	**894**	03/12/2016	94350	D	272	male	no	25/05/2016	T807	positive	1
48	**926**	03/12/2016	77560	NA	816	female	no	28/05/2016	T803	negative	1
49	**987**	04/04/2016	94360	R	1089	male	no	31/05/2016	T800	negative	1
50	**1017**	04/06/2016	94370	D	565	male	no	03/06/2016	T798	negative	2
51	**1048**	04/06/2016	92160	NA	835	male	no	03/06/2016	T799	negative	1
52	**1067**	04/07/2016	78260	D	128	male	no	04/06/2016	T806	negative	7
53	**1069**	04/08/2016	94500	R	175	male	no	04/06/2016	T797	negative	1
54	**1092**	04/09/2016	NA	D	310	NA	no	06/06/2016	T823	negative	5
55	**1093**	04/10/2016	77220	D	324	male	no	06/06/2016	T825	negative	1
56	**1107**	04/11/2016	95180	D	188	female	no	07/06/2016	T824	negative	5
57	**1117**	04/11/2016	78960	D	272	female	no	07/06/2016	T826	negative	1
58	**1131**	04/11/2016	78960	NA	325	male	no	08/06/2016	T827	negative	6
59	**1141**	04/11/2016	94130	NA	541	female	yes	08/06/2016	T829	negative	1
60	**1163**	04/12/2016	94170	D	504	female	yes	09/06/2016	T830	positive	1
61	**1203**	05/08/2016	78130	R	57	male	no	11/06/2016	T865	positive	7
62	**1209**	05/08/2016	93130	E	70	male	no	11/06/2016	T867	negative	5
63	**1210**	05/08/2016	93130	D	75	male	no	13/06/2016	T868	negative	5
64	**1211**	05/09/2016	78670	E	503	male	no	13/06/2016	T864	positive	5
65	**1212**	05/09/2016	92160	R	703	male	yes	11/06/2016	T866	negative	1
66	**1235**	05/10/2016	78180	D	74	female	no	13/06/2016	T863	positive	5
67	**1268**	06/05/2016	28100	D	600	female	no	13/06/2016	T861	negative	1
68	**1269**	06/06/2016	92230	R	212	NA	no	13/06/2016	T859	negative	1
69	**1270**	06/06/2016	75018	D	NA	NA	no	13/06/2016	T860	negative	1
70	**1273**	06/07/2016	94300	D	123	female	no	14/06/2016	T882	negative	1
71	**1281**	06/07/2016	78500	D	196	NA	yes	15/06/2016	T881	negative	1
72	**1313**	06/07/2016	94240	D	200	female	no	13/06/2016	T899	negative	2
73	**1320**	06/07/2016	94350	D	276	NA	no	18/06/2016	T898	negative	2
74	**1341**	06/07/2016	94500	R	668	NA	no	19/06/2016	T900	negative	1
75	**1347**	06/08/2016	92220	R	110	female	no	20/06/2016	T906	negative	2
76	**1348**	06/08/2016	94210	E	872	female	yes	19/06/2016	T897	positive	1
77	**1349**	06/09/2016	91190	D	130	female	no	19/06/2016	T901-1	positive	5
78	**1352**	06/09/2016	93110	E	NA	male	no	19/06/2016	T896	negative	1
79	**1354**	06/10/2016	92230	D	194	female	no	19/06/2016	T908	negative	1
80	**1362**	06/11/2016	77380	NA	224	female	no	19/06/2016	T901-2	negative	2
81	**1363**	06/11/2016	77100	R	406	male	no	19/06/2016	T901-3	negative	3
82	**1364**	06/12/2016	92160	NA	302	female	no	20/06/2016	T910	positive	1
83	**1376**	06/12/2016	91460	D	467	NA	no	20/06/2016	T911	negative	1
84	**1390**	06/12/2016	28130	E	juvenile	female	no	21/06/2016	T909	negative	4

N°	ID	Date of arrival	Postal code	Outcome	Weight at arrival (g)	Sex	Skin lesions	1st sampling date	Sample ID	1st diagnosis	N° of samplings
85	**1405**	07/06/2016	77400	D	51	NA	no	22/06/2016	T925	negative	1
86	**1406**	07/06/2016	NA	R	104	female	no	22/06/2016	T926	negative	1
87	**1410**	07/07/2016	94380	D	80	NA	no	06/07/2016	T932	negative	2
88	**1431**	07/07/2016	78670	D	363	NA	no	23/06/2016	T929	negative	1
89	**1432**	07/07/2016	78960	R	365	female	no	23/06/2016	T930	negative	1
90	**1438**	07/07/2016	92340	D	471	NA	no	23/06/2016	T928	positive	5
91	**1449**	07/10/2016	95880	NA	315	female	no	23/06/2016	T927	negative	1
92	**1454**	07/11/2016	91179	D	juvenile	male	yes	24/06/2016	T955	negative	4
93	**1459**	08/04/2016	92210	D	1000	male	no	24/06/2016	T953	positive	5
94	**1460**	08/05/2016	93440	D	647	male	no	24/06/2016	T952	positive	5
95	**1461**	08/05/2016	NA	E	720	male	no	24/06/2016	T951	positive	5
96	**1466**	08/06/2016	92210	D	91	NA	no	24/06/2016	T954	negative	1
97	**1471**	08/06/2016	95470	NA	119	NA	no	24/06/2016	T948	negative	3
98	**1472**	08/07/2016	91200	R	90	female	no	24/06/2016	T949	negative	3
99	**1473**	08/07/2016	91200	D	98	male	no	24/06/2016	T950	positive	5
100	**1480**	08/08/2016	NA	D	28	female	no	24/06/2016	T947	negative	1
101	**1485**	08/08/2016	95240	D	127	male	no	24/06/2016	T946	negative	1
102	**1492**	08/08/2016	95240	R	135	female	no	25/06/2016	T956	negative	3
103	**1493**	08/08/2016	95240	E	146	male	no	25/06/2016	T957	negative	1
104	**1494**	08/09/2016	94700	D	257	male	no	25/06/2016	T958	negative	1
105	**1497**	08/11/2017	94000	D	208	female	no	25/06/2016	T959	negative	1
106	**1502**	09/06/2016	94260	D	NA	NA	no	26/06/2016	T960	negative	3
107	**1505**	09/07/2016	93100	D	70	female	no	26/06/2016	T961	negative	1
108	**1506**	09/07/2016	95580	D	77	female	no	26/06/2016	T962	negative	3
109	**1510**	09/07/2016	95580	D	79	male	no	26/06/2016	T963	positive	5
110	**1512**	09/07/2016	92410	D	200	female	no	26/06/2016	T964	positive	1
111	**1516**	09/07/2016	NA	R	308	male	no	26/06/2016	T967	positive	3
112	**1517**	09/07/2016	93100	E	529	female	no	26/06/2016	T966	negative	1
113	**1530**	09/09/2016	NA	D	60	female	no	26/06/2016	T968	negative	1
114	**1531**	09/09/2016	92000	D	NA	male	no	26/06/2016	T965	negative	1
115	**1532**	09/10/2016	92330	NA	135	male	no	26/06/2016	T979	negative	2
116	**1537**	09/10/2016	77181	D	217	male	no	27/06/2016	T986	positive	1
117	**1542**	09/10/2016	78150	E	530	male	no	27/06/2016	T984	positive	1
118	**1543**	09/10/2016	94420	E	NA	female	no	27/06/2016	T983	negative	2
119	**1544**	09/10/2016	91600	E	NA	male	no	27/06/2016	T982	negative	1
120	**1545**	09/12/2016	NA	D	242	female	no	27/06/2016	T981	positive	2
121	**1546**	09/12/2016	NA	D	288	female	no	27/06/2016	T980	positive	4
122	**1548**	09/12/2016	NA	D	331	female	no	27/06/2016	T985	negative	1
123	**1589**	10/07/2016	95880	D	58	male	no	29/06/2016	T978	positive	1
124	**1590**	10/07/2016	95880	D	64	female	no	29/06/2016	T1027	negative	1
125	**1596**	10/08/2016	78370	D	117	female	no	29/06/2016	T1021	negative	1
126	**1597**	10/08/2016	78370	D	130	male	yes	29/06/2016	T1020	negative	1
127	**1599**	10/08/2016	78370	R	888	male	no	30/06/2016	T1025	negative	1
128	**1600**	10/09/2016	92110	E	1000	female	no	30/06/2016	T1024	negative	1

N°	ID	Date of arrival	Postal code	Outcome	Weight at arrival (g)	Sex	Skin lesions	1st sampling date	Sample ID	1st diagnosis	N° of samplings
129	**1601**	10/10/2016	94130	NA	266	male	yes	30/06/2016	T1023	negative	1
130	**1602**	10/10/2016	94130	D	298	female	yes	30/06/2016	T1022	negative	1
131	**1606**	10/10/2016	94130	NA	303	female	yes	30/06/2016	T1019	negative	1
132	**1624**	10/10/2016	94130	R	420	male	no	01/07/2016	T1026	positive	1
133	**1640**	10/12/2016	94360	D	339	male	no	01/07/2016	T1017	negative	1
134	**1658**	10/12/2016	91160	NA	459	female	no	02/07/2016	T1016	negative	1
135	**1659**	11/03/2016	78960	D	689	male	yes	02/07/2016	T1015	negative	1
136	**1660**	11/06/2016	75015	NA	105	male	no	02/07/2016	T1014	negative	3
137	**1664**	11/06/2016	95230	R	120	male	no	02/07/2017	T1013	negative	2
138	**1678**	11/06/2016	NA	R	121	female	no	02/07/2016	T1012	negative	1
139	**1680**	11/06/2016	95230	R	125	female	no	02/07/2016	T1010	negative	3
140	**1681**	11/06/2016	92160	D	387	female	no	02/07/2016	T1011	negative	1
141	**1688**	11/07/2016	95100	D	73	female	no	03/07/2016	T1009	negative	3
142	**1696**	11/07/2016	77590	D	juvenile	female	no	03/07/2016	T1029	negative	3
143	**1702**	11/07/2016	94370	D	NA	female	no	03/07/2016	T1008	negative	1
144	**1708**	11/09/2016	94120	NA	142	female	no	04/07/2016	T1038	positive	1
145	**1754**	11/09/2016	94120	D	NA	NA	no	07/07/2016	T1074	positive	1
146	**1761**	11/10/2016	95130	D	127	female	no	06/07/2016	T1078	positive	1
147	**1762**	11/10/2016	94290	D	NA	NA	no	06/07/2016	T1076	positive	1
148	**1765**	11/11/2016	60230	D	120	NA	no	06/07/2016	T1077	negative	1
149	**1775**	11/12/2016	77450	NA	470	female	no	06/07/2016	T1069	negative	1
150	**1780**	12/04/2016	93160	D	524	male	no	07/07/2016	T1080	negative	1
151	**1782**	12/04/2016	51100	E	NA	female	no	07/07/2016	T1072	positive	1
152	**1793**	12/05/2016	75005	E	557	NA	yes	07/07/2016	T1092	positive	1
153	**1795**	12/06/2016	NA	R	121	male	no	07/07/2016	T1081	negative	1
154	**1809**	12/07/2016	91160	E	700	NA	no	08/07/2016	T1071	positive	1
155	**1810**	12/07/2016	75017	R	1020	male	no	08/07/2016	T1062	negative	3
156	**1819**	12/07/2016	92220	D	NA	NA	no	09/07/2016	T1093	negative	1
157	**1820**	12/08/2016	NA	R	127	male	no	09/07/2016	T1095	positive	1
158	**1821**	12/08/2016	77380	E	NA	female	no	09/07/2016	T1094	positive	1
159	**1828**	12/09/2016	94400	E	110	NA	no	09/07/2016	T1096	negative	1
160	**1829**	12/09/2016	94400	NA	116	NA	no	09/07/2016	T1082	negative	1
161	**1832**	12/09/2016	94400	D	121	female	no	09/07/2016	T1067	negative	2
162	**1850**	12/10/2016	77181	D	223	male	no	10/07/2016	T1097	negative	1
163	**1851**	12/10/2016	94800	NA	230	male	no	10/07/2016	T1068	positive	1
164	**1865**	12/10/2016	77120	NA	274	NA	no	10/07/2016	T1083	positive	1
165	**1893**	12/10/2016	95000	D	284	female	yes	11/07/2016	T1099	negative	1
166	**1894**	12/10/2016	NA	D	NA	NA	no	11/07/2016	T1101	negative	1
167	**1910**	12/11/2016	78330	NA	377	NA	no	12/07/2016	T1064	negative	2
168	**1914**	12/11/2016	60500	D	406	female	no	13/07/2016	T1066	positive	1
169	**1915**	13/02/2016	75018	R	697	female	no	13/07/2016	T1100	negative	2
170	**1916**	13/02/2016	94140	R	1001	male	no	13/07/2016	T1065	negative	2
171	**1917**	13/04/2016	94350	D	503	male	no	13/07/2016	T1079	negative	1
172	**1918**	13/05/2016	94100	R	830	male	no	13/07/2016	T1063	positive	1

N°	ID	Date of arrival	Postal code	Outcome	Weight at arrival (g)	Sex	Skin lesions	1st sampling date	Sample ID	1st diagnosis	N° of samplings
173	**1927**	13/06/2016	92410	D	82	male	no	14/07/2016	T1139	positive	1
174	**1941**	13/06/2016	78100	D	150	NA	no	14/07/2016	T1138	negative	1
175	**1945**	13/06/2016	78100	D	172	male	no	14/07/2016	T1134	negative	1
176	**1946**	13/06/2016	75012	R	313	male	no	14/07/2016	T1137	negative	1
177	**1947**	13/08/2016	95230	D	100	female	no	14/07/2016	T1136	negative	1
178	**1951**	13/08/2016	95230	R	108	NA	no	15/07/2016	T1135	negative	2
179	**1954**	13/08/2016	95230	D	126	male	no	15/07/2016	T1130	positive	2
180	**1955**	13/08/2016	95230	D	130	female	no	15/07/2016	T1131	negative	2
181	**1957**	13/08/2016	94440	R	350	female	no	15/07/2016	T1133	negative	2
182	**1958**	13/08/2016	77200	D	440	NA	no	15/07/2016	T1129	negative	1
183	**1962**	13/08/2016	NA	D	NA	NA	yes	16/07/2016	T1122	negative	1
184	**1990**	13/10/2016	75012	D	210	male	no	17/07/2016	T1126	negative	3
185	**1991**	13/10/2016	NA	D	313	NA	no	17/07/2016	T1132	negative	1
186	**2003**	13/11/2016	91080	D	506	female	no	17/07/2016	T1128	negative	1
187	**2011**	14/05/2016	94700	D	440	female	no	17/07/2016	T1127	negative	1
188	**2014**	14/06/2016	77090	D	109	female	yes	18/07/2016	T1125	negative	1
189	**2020**	14/07/2016	95100	D	120	female	no	18/07/2016	T1124	negative	1
190	**2022**	14/07/2016	94700	D	385	female	no	18/07/2016	T1123	negative	1
191	**2074**	14/07/2016	94700	D	483	male	no	20/07/2016	T1160	negative	2
192	**2085**	14/07/2016	77370	D	NA	NA	no	20/07/2016	T1161	negative	1
193	**2096**	14/09/2016	93420	D	99	female	no	21/07/2016	T1158	negative	1
194	**2106**	14/09/2016	93420	NA	103	female	no	21/07/2016	T1159	negative	1
195	**2111**	14/09/2016	93420	NA	103	female	no	21/07/2016	T1157	positive	1
196	**2158**	14/10/2016	77170	D	187	male	no	25/07/2016	T1188	negative	2
197	**2166**	15/04/2016	77340	R	854	female	no	25/07/2016	T1190	positive	4
198	**2169**	15/04/2016	77340	R	1076	male	no	25/07/2016	T1189	negative	2
199	**2171**	15/05/2016	NA	D	470	male	no	25/07/2016	T1185	negative	2
200	**2173**	15/06/2016	94360	D	335	NA	no	26/07/2016	T1186	negative	1
201	**2174**	15/07/2016	95530	R	130	female	no	26/07/2016	T1187	negative	1
202	**2181**	15/07/2016	94430	R	221	female	no	25/07/2016	T1184	positive	4
203	**2201**	15/07/2016	92160	R	680	female	no	26/07/2016	T1183	negative	1
204	**2207**	15/07/2016	77170	R	953	female	no	27/07/2016	T1182	negative	1
205	**2220**	15/07/2016	78670	D	NA	female	no	27/07/2016	T1180	negative	1
206	**2221**	15/08/2016	77310	E	10	NA	yes	27/07/2016	T1181	negative	1
207	**2222**	15/08/2016	77310	E	14	male	yes	27/07/2016	T1179	negative	1
208	**2225**	15/08/2016	77310	E	15	female	no	27/07/2016	T1177	negative	2
209	**2236**	15/09/2016	94440	D	340	male	no	28/07/2016	T1178	positive	1
210	**2269**	15/11/2016	78800	D	421	female	no	01/08/2016	T1229	negative	3
211	**2270**	15/12/2016	78340	NA	330	male	no	01/08/2016	T1228	negative	3
212	**2271**	16/07/2016	91430	D	528	male	no	01/08/2016	T1214	negative	3
213	**2272**	16/08/2016	77750	E	NA	NA	no	30/07/2016	T1260	negative	4
214	**2274**	16/09/2016	60100	D	173	NA	no	30/07/2016	T1221	negative	1
215	**2295**	16/10/2016	94000	D	178	male	no	31/07/2016	T1223	negative	2
216	**2301**	16/10/2016	77610	D	218	female	no	31/07/2016	T1224	negative	1

N°	ID	Date of arrival	Postal code	Outcome	Weight at arrival (g)	Sex	Skin lesions	1st sampling date	Sample ID	1st diagnosis	N° of samplings
217	**2308**	16/10/2016	NA	D	297	male	no	31/07/2016	T1225	positive	1
218	**2336**	16/10/2016	NA	D	347	male	no	02/08/2016	T1261	negative	1
219	**2339**	16/11/2016	77181	D	253	male	yes	03/08/2016	T1263	negative	2
220	**2349**	16/11/2016	77700	D	278	male	yes	03/08/2016	T1252	negative	1
221	**2351**	16/11/2016	NA	NA	NA	NA	yes	03/08/2016	T1268	negative	2
222	**2358**	17/04/2016	93270	E	656	NA	no	04/08/2016	T1247	negative	3
223	**2361**	17/05/2016	94700	D	158	female	no	05/08/2016	T1246	negative	3
224	**2363**	17/07/2016	93250	R	77	female	no	05/08/2016	T1267	negative	3
225	**2371**	17/07/2016	94310	D	107	male	no	05/08/2016	T1259	negative	1
226	**2375**	17/07/2016	91550	D	152	NA	no	06/08/2016	T1258	negative	1
227	**2378**	17/07/2016	77200	D	adult	NA	no	06/08/2016	T1257	negative	3
228	**2396**	17/08/2016	75019	E	90	female	no	08/08/2016	T1245	negative	1
229	**2398**	17/08/2016	94500	D	164	NA	no	08/08/2016	T1242	negative	3
230	**2399**	17/10/2016	95100	R	749	male	no	08/08/2016	T1243	negative	4
231	**2400**	17/10/2016	95100	D	NA	NA	no	08/08/2016	T1244	negative	1
232	**2416**	17/10/2016	75020	D	NA	NA	no	10/08/2016	T1254	positive	1
233	**2417**	18/04/2016	94380	D	454	male	no	10/08/2016	T1256	positive	1
234	**2418**	18/05/2016	94400	D	510	male	yes	10/08/2016	T1262	negative	1
235	**2442**	18/05/2016	91330	E	adult	female	no	12/08/2016	T1341	negative	3
236	**2444**	18/06/2016	92350	R	95	NA	no	12/08/2016	T1367	negative	1
237	**2452**	18/07/2016	28500	E	95	NA	no	15/08/2016	T1421	negative	1
238	**2453**	18/07/2016	78150	D	224	male	no	13/08/2016	T1356	negative	3
239	**2454**	18/07/2016	94130	D	NA	NA	no	13/08/2016	T1398	negative	2
240	**2459**	18/09/2016	91380	E	268	NA	no	13/08/2016	T1365	negative	1
241	**2460**	18/09/2016	95480	D	BB	NA	no	13/08/2016	T1400	negative	1
242	**2461**	18/09/2016	95480	D	BB	NA	no	13/08/2016	T1399	negative	1
243	**2462**	18/10/2016	NA	NA	148	male	no	13/08/2016	T1401	negative	1
244	**2475**	18/10/2016	NA	NA	260	male	no	15/08/2016	T1402	negative	1
245	**2476**	18/10/2016	NA	D	270	female	no	15/08/2016	T1403	negative	1
246	**2477**	18/10/2016	94290	D	adult	female	no	15/08/2016	T1404	negative	1
247	**2487**	18/12/2016	92160	NA	385	female	no	16/08/2016	T1371	negative	1
248	**2492**	18/12/2016	77400	NA	428	female	no	17/08/2016	T1415	positive	1
249	**2496**	19/04/2016	92160	E	549	male	no	17/08/2016	T1418	positive	1
250	**2515**	19/06/2016	94170	E	113	NA	no	19/08/2016	T1416	negative	1
251	**2516**	19/06/2016	92190	D	124	male	no	19/08/2016	T1417	positive	1
252	**2524**	19/06/2016	92190	E	125	male	no	20/08/2016	T1390	negative	1
253	**2527**	19/06/2016	77220	D	147	male	yes	20/08/2016	T1389	negative	1
254	**2534**	19/06/2016	92190	R	149	male	no	21/08/2016	T1351	positive	4
255	**2535**	19/06/2016	77178	D	460	NA	no	21/08/2016	T1340	positive	6
256	**2536**	19/06/2016	60500	D	550	NA	no	21/08/2016	T1350	positive	4
257	**2543**	19/06/2016	95170	R	686	female	no	21/08/2016	T1387	negative	1
258	**2565**	19/08/2016	91400	D	577	female	no	23/08/2016	T1383	negative	1
259	**2566**	19/08/2016	94000	E	NA	female	no	23/08/2016	T1409	negative	1
260	**2567**	20/06/2016	94420	D	460	male	no	23/08/2016	T1384	negative	1

N°	ID	Date of arrival	Postal code	Outcome	Weight at arrival (g)	Sex	Skin lesions	1st sampling date	Sample ID	1st diagnosis	N° of samplings
261	**2572**	20/06/2016	60330	D	600	female	no	24/08/2016	T1412	negative	1
262	**2580**	20/07/2016	94500	D	101	male	no	24/08/2016	T1353	negative	2
263	**2581**	20/07/2016	94410	R	403	male	no	24/08/2016	T1352	negative	1
264	**2598**	20/08/2016	78180	E	50	NA	no	26/08/2016	T1411	positive	3
265	**2599**	20/08/2016	91700	D	880	female	no	26/08/2016	T1363	negative	1
266	**2600**	20/09/2016	75016	D	NA	female	no	26/08/2016	T1408	negative	6
267	**2601**	20/11/2016	95180	NA	290	male	no	26/08/2016	T1346	negative	1
268	**2602**	20/11/2016	91540	D	393	female	no	26/08/2016	T1347	negative	1
269	**2638**	20/11/2016	77330		539	female	no	29/08/2016	T1326	positive	1
270	**2639**	21/05/2016	94440	R	1400	female	no	29/08/2016	T1325	positive	4
271	**2642**	21/06/2016	94290	R	101	NA	no	29/08/2016	T1379	positive	4
272	**2647**	21/07/2016	78740	D	85	female	no	31/08/2016	T1377	positive	1
273	**2649**	21/07/2016	78740	E	103	male	yes	31/08/2016	T1378	negative	2
274	**2650**	21/07/2016	77590	D	NA	male	no	30/08/2016	T1424	negative	1
275	**2678**	21/08/2016	95410	NA	151	NA	no	01/09/2016	T1344	negative	2
276	**2688**	21/08/2016	95410	NA	156	NA	no	02/09/2016	T1345	negative	1
277	**2695**	21/08/2016	95410	NA	162	female	no	02/09/2016	T1462	negative	1
278	**2700**	21/08/2016	91200	D	247	NA	yes	03/09/2016	T1349	positive	1
279	**2702**	21/09/2016	94160	E	291	female	no	03/09/2016	T1339	positive	1
280	**2703**	21/09/2016	77184	D	294	NA	no	03/09/2016	T1337	positive	5
281	**2704**	21/09/2016	93330	NA	296	male	no	03/09/2016	T1426	positive	4
282	**2705**	21/09/2016	94320	R	582	NA	no	03/09/2016	T1364	negative	3
283	**2711**	21/11/2016	78330	D	222	female	no	03/09/2016	T1405	positive	1
284	**2729**	21/11/2016	60500	D	366	male	no	04/09/2016	T1459	negative	1
285	**2735**	22/04/2016	94550	R	544	female	no	05/09/2016	T1458	positive	1
286	**2736**	22/05/2016	94320	E	409	male	no	05/09/2016	T1457	negative	1
287	**2750**	22/06/2016	94700	D	NA	NA	no	06/09/2016	T1464	negative	1
288	**2752**	22/06/2016	92000	D	NA	NA	no	06/09/2016	T1463	negative	1
289	**2778**	22/10/2016	94190	R	958	male	no	08/09/2016	T1456	negative	3
290	**2781**	22/10/2016	94250	E	adult	female	no	09/09/2016	T1455	negative	1
291	**2783**	22/11/2016	94500	E	214	male	yes	09/09/2016	T1454	negative	1
292	**2799**	23/04/2016	94450	R	689	male	no	10/09/2016	T1453	negative	1
293	**2817**	23/06/2016	78350	D	96	male	no	11/09/2016	T1451	positive	4
294	**2818**	23/06/2016	78350	D	103	male	no	11/09/2016	T1452	positive	1
295	**2824**	23/06/2016	95190	R	133	male	no	12/09/2016	T1448	negative	3
296	**2825**	23/06/2016	91790	E	517	male	no	12/09/2016	T1449	negative	1
297	**2826**	23/06/2016	94430	D	599	male	no	12/09/2016	T1450	negative	2
298	**2839**	23/08/2016	78350	E	91	male	no	14/09/2016	T1489	negative	1
299	**2840**	23/08/2016	78350	E	95	male	no	15/09/2016	T1488	negative	4
300	**2841**	23/08/2016	78350	E	97	female	no	14/09/2016	T1490	negative	1
301	**2874**	23/11/2016	94700	D	443	female	no	15/09/2016	T1502	negative	2
302	**2881**	24/05/2016	94130	R	540	female	no	16/09/2016	T1503	negative	1
303	**2895**	24/06/2016	78860	D	96	male	no	18/09/2016	T1507	negative	1
304	**2896**	24/06/2016	93700	R	99	male	no	18/09/2016	T1501	positive	1

N°	ID	Date of arrival	Postal code	Outcome	Weight at arrival (g)	Sex	Skin lesions	1st sampling date	Sample ID	1st diagnosis	N° of samplings
305	**2897**	24/06/2016	95100	R	100	male	no	18/09/2016	T1501b	negative	1
306	**2914**	24/06/2016	93700	R	101	male	no	20/09/2016	T1538	negative	1
307	**2915**	24/06/2016	95100	R	107	female	no	21/09/2016	T1572	negative	1
308	**2919**	24/06/2016	94800	E	107	male	no	21/09/2016	T1571	positive	1
309	**2921**	24/06/2016	93700	R	110	male	no	21/09/2016	T1570	negative	3
310	**2925**	24/06/2016	28700	D	488	male	no	21/09/2016	T1568	negative	1
311	**2961**	24/06/2016	92700	R	524	male	no	25/09/2016	T1579	negative	2
312	**2974**	24/06/2016	95100	R	875	male	yes	26/09/2016	T1638	negative	1
313	**3004**	24/08/2016	77450	D	200	female	no	30/09/2016	T1640	negative	1
314	**3006**	24/08/2016	77450	D	200	female	no	30/09/2016	T1636	negative	1
315	**3019**	24/08/2016	93250	D	623	female	no	02/10/2016	T1637	negative	3
316	**3024**	24/10/2016	77860	R	172	male	no	02/10/2016	T1668	negative	1
317	**3026**	24/10/2016	60500	D	414	male	no	03/10/2016	T1667	positive	5
318	**3032**	25/10/2016	28290	D	358	male	no	04/10/2016	T1669	positive	1
319	**3043**	25/03/2016	77176	R	750	female	yes	05/10/2016	T1681	negative	1
320	**3050**	25/04/2016	94500	R	700	male	yes	07/10/2016	T1682	negative	1
321	**3054**	25/05/2016	92370	R	770	female	no	07/10/2016	T1670	positive	3
322	**3062**	25/06/2016	60240	D	80	male	no	09/10/2016	T1680	positive	1
323	**3063**	25/06/2016	60240	D	90	male	no	09/10/2016	T1666	negative	3
324	**3067**	25/06/2016	60240	R	125	female	no	09/10/2016	T1679	negative	1
325	**3068**	25/06/2016	95500	D	496	NA	no	09/10/2016	T1678	negative	1
326	**3071**	25/07/2016	NA	R	430	female	no	09/10/2016	T1677	negative	1
327	**3080**	25/07/2016	NA	D	430	male	no	10/10/2016	T1743	negative	1
328	**3081**	25/07/2016	78140	R	445	female	no	10/10/2016	T1751	negative	2
329	**3082**	25/07/2016	NA	R	532	male	no	10/10/2016	T1741	negative	2
330	**3083**	25/07/2016	93340	R	694	male	no	10/10/2016	T1675	negative	3
331	**3090**	25/08/2016	91600	NA	76	female	no	11/10/2016	T1676	negative	1
332	**3092**	25/09/2016	95230	NA	163	female	no	11/10/2016	T1760	negative	1
333	**3095**	26/04/2016	94100	D	579	female	yes	12/10/2016	T1744	negative	1
334	**3097**	26/06/2016	78700	R	85	female	no	12/10/2016	T1771	negative	1
335	**3100**	26/06/2016	92250	R	102	female	no	12/10/2016	T1742	positive	3
336	**3101**	26/06/2016	91090	R	109	female	no	12/10/2016	T1770	negative	1
337	**3103**	26/06/2016	NA	D	110	male	no	13/10/2016	T1740	negative	3
338	**3107**	26/06/2016	95150	R	113	male	no	13/10/2016	T1745	negative	2
339	**3109**	26/06/2016	NA	D	159	NA	no	13/10/2016	T1746	negative	1
340	**3116**	26/06/2016	94520	R	759	female	no	14/10/2016	T1755	negative	1
341	**3129**	26/06/2016	77420	E	114	male	no	16/10/2016	T1747	negative	1
342	**3133**	26/06/2016	78700	D	NA	female	no	16/10/2016	T1739	negative	1
343	**3135**	26/06/2016	93000	E	546	NA	no	16/10/2016	T1748	negative	1
344	**3136**	26/07/2016	78500	D	65	male	no	16/10/2016	T1749	negative	1
345	**3144**	26/07/2016	78500	D	70	male	no	17/10/2016	T1750	negative	2
346	**3147**	26/07/2016	92330	D	NA	NA	no	17/10/2016	T1761	negative	1
347	**3150**	26/08/2016	91600	D	66	female	no	17/10/2016	T1759	negative	1
348	**3155**	26/08/2016	91600	NA	80	male	no	18/10/2016	T1758	negative	1

N°	ID	Date of arrival	Postal code	Outcome	Weight at arrival (g)	Sex	Skin lesions	1st sampling date	Sample ID	1st diagnosis	N° of samplings
349	**3156**	26/08/2016	94140	D	790	male	no	18/10/2016	T1738	negative	1
350	**3157**	26/08/2016	94140	E	840	male	no	18/10/2016	T1737	negative	3
351	**3158**	26/09/2016	92380	E	111	NA	no	18/10/2016	T1736	negative	3
352	**3182**	27/06/2016	95740	D	40	male	no	22/10/2016	T1883	negative	1
353	**3185**	27/06/2016	77220	D	150	female	no	22/10/2016	T1839	negative	1
354	**3189**	27/06/2016	77220	D	165	female	no	24/10/2016	T1841	negative	1
355	**3193**	27/06/2016	77220	D	170	female	no	24/10/2016	T1840	negative	2
356	**3212**	27/06/2016	77220	R	191	female	no	27/10/2016	T1837	negative	1
357	**3213**	27/06/2016	27700	E	575	female	no	27/10/2016	T1834	negative	3
358	**3214**	27/06/2016	94160	E	602	NA	no	27/10/2016	T1838	positive	2
359	**3216**	27/07/2016	92190	R	312	female	no	27/10/2016	T1884	negative	1
360	**3222**	27/07/2016	NA	D	650	female	no	28/10/2016	T1835	negative	1
361	**3223**	27/07/2016	78510	D	960	male	no	28/10/2016	T1836	negative	2
362	**3224**	27/07/2016	92370	D	adult	NA	no	28/10/2016	T1882	negative	1
363	**3228**	27/07/2016	78320	D	260	NA	no	29/10/2016	T1833	negative	1
364	**3253**	27/10/2016	95270	D	210	female	no	02/11/2016	T1865	negative	1
365	**3258**	27/10/2016	95270	NA	264	male	no	28/10/2016	T1877	negative	1
366	**3267**	27/10/2016	77000	E	323	female	no	04/11/2016	T1867	negative	2
367	**3268**	27/10/2016	95270	NA	340	female	no	04/11/2016	T1868	negative	2
368	**3269**	28/04/2016	95170	E	589	male	no	04/11/2016	T1873	negative	1
369	**3271**	28/05/2016	91180	E	adult	female	no	04/11/2016	T1866	negative	1
370	**3283**	28/07/2016	77000	D	208	female	no	06/11/2016	T1930	negative	2
371	**3284**	28/10/2016	93200	D	188	NA	no	06/11/2016	T1942	negative	1
372	**3287**	28/10/2016	94100	D	197	female	no	07/11/2016	T1929	positive	1
373	**3290**	28/10/2016	94100	NA	221	male	no	08/11/2017	T1941	negative	1
374	**3304**	28/11/2016	60230	D	147	female	no	11/11/2016	T1963	negative	1
375	**3306**	28/11/2016	93290	NA	559	male	no	12/11/2016	T1965	negative	1
376	**3310**	28/11/2016	94420	D	juvenile	male	no	12/11/2016	T1964	negative	2
377	**3317**	29/03/2016	NA	R	503	female	no	13/11/2016	T1962	negative	1
378	**3326**	29/04/2016	92600	D	428	female	no	15/11/2016	T1966	negative	1
379	**3328**	29/04/2016	77122	D	544	male	yes	16/11/2016	T1969	positive	5
380	**3330**	29/06/2016	95520	E	93	male	no	16/11/2016	T1968	positive	1
381	**3332**	29/06/2016	NA	R	542	male	no	16/11/2016	T1961	positive	1
382	**3347**	29/06/2016	95800	D	580	male	yes	20/11/2016	T1999	positive	2
383	**3348**	29/06/2016	NA	R	592	female	no	20/11/2016	T1998	positive	3
384	**3352**	29/08/2016	91600	NA	63	male	no	20/11/2016	T1997	positive	1
385	**3353**	29/08/2016	95210	D	95	female	yes	21/11/2016	T2008	positive	2
386	**3357**	29/08/2016	95210		138	male	yes	21/11/2016	T2009	negative	1
387	**3359**	29/10/2016	91420	D	632	male	no	22/11/2016	T2153	positive	1
388	**3361**	29/11/2016	78570	D	305	female	no	23/11/2016	T2152	negative	1
389	**3380**	30/03/2016	95420	R	974	male	no	28/11/2016	T2099	negative	1
390	**3381**	30/06/2016	NA	D	37	NA	no	28/11/2016	T2151	negative	1
391	**3385**	30/06/2016	NA	D	44	NA	no	28/11/2016	T2098	negative	1
392	**3391**	30/06/2016	NA	D	47	NA	no	29/11/2016	T2097	negative	1

N°	ID	Date of arrival	Postal code	Outcome	Weight at arrival (g)	Sex	Skin lesions	1st sampling date	Sample ID	1st diagnosis	N° of samplings
393	**3397**	30/06/2016	NA	D	49	NA	no	30/11/2016	T2096	positive	3
394	**3398**	30/06/2016	72340	D	63	NA	no	30/11/2016	T2094	positive	3
395	**3399**	30/06/2016	91140	R	1095	male	no	30/11/2016	T2093	negative	1
396	**3404**	30/07/2016	60660	R	92	NA	no	01/12/2016	T2092	negative	1
397	**3408**	30/07/2016	60660	R	94	male	no	02/12/2016	T2150	negative	1
398	**3409**	30/07/2016	60660	NA	97	female	no	03/12/2016	T2091	negative	1
399	**3414**	30/07/2016	60660	R	98	female	no	03/12/2016	T2090	negative	1
400	**3415**	30/07/2016	94700	D	328	NA	no	04/12/2016	T2089	positive	1
401	**3419**	30/08/2016	95320	D	45	male	no	06/12/2016	T2095	negative	1
402	**3420**	30/08/2016	95320	R	48	female	no	06/12/2016	T2154	negative	1
403	**3421**	30/08/2016	94130	R	380	NA	no	06/12/2016	T2088	negative	1
404	**3435**	30/09/2016	94200	D	119	NA	no	09/12/2016	T2148	negative	1
405	**3436**	30/09/2016	95140	E	216	NA	no	09/12/2016	T2149	negative	1
406	**3437**	30/11/2016	95390	NA	229	male	no	09/12/2016	T2147	negative	1
407	**3440**	30/11/2016	95390	NA	288	female	yes	10/12/2016	T2146	positive	1
408	**3442**	30/11/2016	95110	R	NA	male	yes	10/12/2016	T2145	negative	1
409	**3444**	31/05/2016	78280	D	464	male	no	11/12/2016	T2144	negative	1
410	**3452**	31/07/2016	91700	R	650	male	no	15/12/2016	T2188	negative	1
411	**3458**	31/07/2016	94000	E	895	male	no	18/12/2016	T2189	negative	1
412	**3460**	31/07/2016	94700	R	1100	female	yes	18/12/2016	T2190	positive	2
